# Acid Resistance in *Francisella tularensis*

**DOI:** 10.1002/mbo3.151

**Published:** 2014-01-08

**Authors:** Noreen J Adcock, Brian J Morris, Eugene W Rice

**Affiliations:** 1 U.S. Environmental Protection AgencyCincinnati, Ohio; 2 Pegasus Technical Services Inc.Cincinnati, Ohio

**Keywords:** Gastric fluid, ingestion, tularemia, waterborne

## Abstract

*Francisella tularensis*, the etiologic agent of tularemia, can survive under acidic conditions. Tularemia can be acquired by several routes, including by ingestion of contaminated food or water. While acid resistance is usually associated with a low oral infective dose (ID), the ID for gastrointestinal illness is quite high. In this study, four strains of *F. tularensis* ssp. *tularensis* (type A) and four strains of *F. tularensis* ssp. *holarctica* (type B) were examined for innate acid resistance and the ability to survive in synthetic gastric fluid (SGF) under in vitro conditions similar to passage through the human stomach. Survival for all strains was significantly less in pH 2.5 SGF than in pH 2.5 phosphate-buffered saline and pH 4.0 SGF. Attenuated strains were consistently less resistant. Type B strains are most often associated with waterborne outbreaks and were examined after storage in natural water. Low-nutrient preadaptation resulted in increased resistance. Although *F. tularensis* can persist under certain acidic conditions, it is sensitive to conditions replicating the fasting human stomach. This may help explain the high ID required for gastrointestinal infections.

## Introduction

*Francisella tularensis*, a gram-negative, facultative, intracellular bacterium, is the etiologic agent of the zoonotic disease tularemia. The organism has also been considered as a potential biological weapon (Dennis et al. 2001). *F. tularensis* ssp. *tularensis* (type A) and *F. tularensis* ssp. *holarctica* (type B) are the two most virulent forms associated with human infections. The pathogen has a wide host range and can be spread by the bite of an arthropod, direct contact with diseased animals, inhalation, or ingestion of contaminated food or water. Different clinical presentations are associated with the mode of transmission. Infection due to ingestion most commonly results in the oropharyngeal form of tularemia, which is characterized by pharyngitis and cervical lymphadenopathy. Gastrointestinal disease, which may include abdominal pain, nausea, vomiting, diarrhea, and gastrointestinal bleeding, can also be acquired by ingestion, but is extremely rare. Water has served as the major vehicle of transmission for large-scale outbreaks acquired by the oral route, whereas foodborne infections are often associated with inadequate cooking procedures. Waterborne tularemia was first recognized over 75 years ago (Karpoff and Antononoff 1936) and continues to be a public health concern in many areas (Akalin et al. 2009; Grunow et al. 2012). *F. tularensis* ssp. *holarctica* is most often associated with waterborne outbreaks, whereas both subspecies may be responsible for foodborne illness.

Low gastric pH is believed to act as a barrier to infection due to ingestion (Zhu et al. 2006). An extreme acid resistance system is present in *Francisella*, but the genetic basis for this system has not been clearly defined (Rohmer et al. 2006). Resistance of *F. tularensis* to low-pH conditions in vitro has been reported (KuoLee et al. 2007) and acid pretreatment has been proposed as a method for detection of *F. tularensis* in water (Humrighouse et al. 2011). Unlike some enteric pathogens where acid resistance has been associated with a low ID, the oral ID for *F. tularensis* gastrointestinal infections is quite high (Hornick et al. 1967). Although some enteric pathogens exhibit an inducible acid tolerance associated with prior exposure to a mildly acidic pH (Small 1994), this response is unlikely to play a role in gastric survival for *F. tularensis* acquired by ingestion of contaminated water. In this study, acid resistance was determined for both type A and type B virulent and attenuated strains of *F. tularensis*. The role of preadaptation in a low-nutrient aquatic environment prior to acid exposure was evaluated in relation to the role of this agent as a waterborne pathogen.

## Material and Methods

### Bacteria and growth conditions

Three virulent strains of *F. tularensis* ssp. *tularensis* (type A1) and three virulent strains of *F. tularensis* ssp. *holarctica* (type B), originally collected from various geographical locations in the United States, were used in this study. Two attenuated strains, *F. tularensis* ssp. *tularensis* KC1482 (aka ATCC 6223) and *F. tularensis* ssp. *holarctica* live vaccine strain (LVS), were also included (Table 1). Cultures were stored at −80°C in brain heart infusion broth with 15% (v/v) glycerol. A thawed suspension of individual frozen stock cultures was transferred to tryptic soy broth containing 2% (v/v) IsoVitaleX™ (Becton Dickinson, Sparks, MD) and incubated at 37°C for 48 h for each experiment. Stationary-phase cultures were concentrated by centrifugation (3000*g* for 10 min at 4°C) and resuspended in sterile, pH 7.4, phosphate-buffered saline (PBS).

**Table 1:** *Francisella tularensis* strains used in this study.

Subspecies and type designation	Isolate/Origin
*F. tularensis* ssp. *tularensis* (type A1)	F2246/Maryland
*F. tularensis* ssp. *tularensis* (type A1)	H3563/Oklahoma
*F. tularensis* ssp. *tularensis* (type A1)	SchuS4/Ohio
*F. tularensis* ssp. *tularensis* (type A2, attenuated)	KC1482^1^/Utah
*F. tularensis* ssp. *holarctica* (type B)	IN99/Indiana
*F. tularensis* ssp. *holarctica* (type B)	NY98/New York
*F. tularensis* ssp. *holarctica* (type B)	OR96/Oregon
*F. tularensis* ssp. *holarctica* (type B, attenuated)	LVS

LVS, live vaccine strain.

1 KC1482 is denoted as ATCC 6223 in Broekhuijsen et al. 2003.

### Acid exposure

Cell suspensions were centrifuged as described earlier and resuspended in one of three acidic solutions. The synthetic gastric fluid (SGF) formulation, which was that of Beumer et al. (1992) as modified by Casey et al. (2004) by the omission of proteose peptone, was used to replicate conditions which might be found in the human stomach. Acidic PBS was used to examine innate acid resistance. SGF exposures were at pH 2.5 and 4.0 and PBS exposure was at pH 2.5. A pH of 2.5 was chosen as being representative of the human median luminal gastric pH under fasting conditions (Gorden and Small 1993). Exposure in SGF (pH 4.0) was chosen as indicative of the potential buffering effect of food on gastric acidity (Zhu et al. 2006). All solutions were prewarmed to 37°C. The cell suspensions were diluted to an initial titer of 6.0 ± 0.5 log_10_ CFU/mL. Because human gastric emptying times can be influenced by a variety factors, such as liquid or solid intake, gender, and age, exposures were conducted over a series of timed intervals (30, 60, 90, and 120 min) to reflect these differences (Smith 2003). Four tubes of each acidic suspension representing the four exposure times and one tube of a suspension in pH 7.4 PBS were incubated at 37°C. The pH neutral suspensions served as the control suspensions and were assayed at 0 and 120 min and varied by <0.2 log_10_ CFU/mL during the course of the experiments. At the appropriate target incubation times, suspensions were immediately diluted in PBS (pH 7.4) and assayed by the spread plate procedure using Chocolate II agar incubated at 37°C for 6 days. All tests were performed in triplicate using duplicate plates for each dilution. Experiments with virulent strains were conducted by Select Agent Program (U.S. Centers for Disease Control and Prevention) approved analysts under biosafety level 3 (BSL3) conditions at the University of Cincinnati, College of Medicine. All protocols were approved by the university institutional biological safety committee.

### Acid exposure after storage in water

The *F. tularensis* ssp. *holarctica* (type B) strains were grown as described earlier, centrifuged and resuspended in pH 7.4 PBS three times and then resuspended in natural water samples diluted so that the initial titer was 5.8 ± 0.8 log_10_ CFU/mL in 24 mL. The water samples were collected from a local creek, sterilized by autoclaving and stored at 5°C for less than a week prior to use. *F. tularensis* cells were exposed to low-nutrient conditions by suspension in sterile creek water (mean pH, 8.70; turbidity, 11.1 ntu; alkalinity, 114 mg/L; and total organic carbon, 8.58 mg/L) for 24 h at 5°C. Nonnutrient stressed cells were grown under the conditions cited earlier in this article. The stressed and nonstressed cells were compared based upon their response to acid exposure in SGF (pH 2.5) for 120 min. Only type B strains were examined as this is the predominate subspecies which has been associated with waterborne outbreaks. All tests were performed in duplicate.

### Statistical analysis

Percent survival was calculated using the formula: 100 − [(*T*_0_ − *T*_*n*_)/*T*_0_ × 100], where *T*_0_ is the initial titer and *T*_*n*_ is the titer for a given exposure time in CFU/mL. Strains demonstrating greater than 10% survival after 2 h exposure were considered acid resistant (Gorden and Small 1993).

A linear regression model predicting the log survival over time was fitted to the experimental data from the three acidic exposure conditions. The best fit model was a function of the characterization of the *F. tularensis* strain (virulent or attenuated) and the type of acidic conditions (pH 2.5 SGF, pH 4.0 SGF, and pH 2.5 PBS). From the covariance matrix produced by this model, pair-wise two-sided *t*-tests were performed for the three acid exposure conditions. For comparisons between results for cells with low-nutrient preexposure in sterile creek water and cells which were not nutrient stressed, a two-way analysis of variance (ANOVA) model was developed as a function of bacterial strain and the presence or absence of nutrient stress. From the covariance matrix produced by this model, *t*-tests were performed for each strain. A significance level of 0.05 was used to determine statistical significance for all tests.

## Results

### Acid exposure

**Figure 1:**
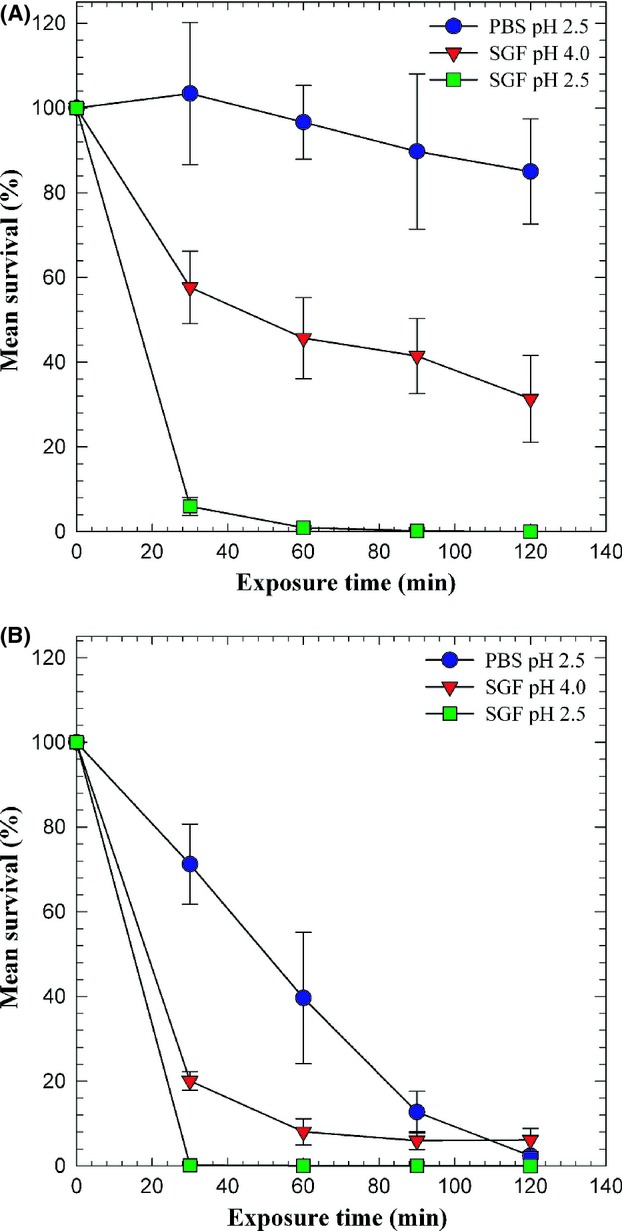
Mean percent survival of *Francisella tularensis* isolates exposed to pH 2.5 and pH 4.0 synthetic gastric fluid (SGF) and pH 2.5 phosphate-buffered saline (PBS). Data represent the means ± standard deviations of three independent experiments for each of (A) six virulent strains and (B) two attenuated strains.

Results showing differences in response to the various acidic conditions for the virulent and attenuated strains are shown in Figure 1. There was no statistically significant difference in response between individual strains from the two groups (*P* = 0.528). Using the 10% survival criterion after 120 min exposure, none of the isolates exposed to SGF (pH 2.5) would be characterized as acid resistant. After 60 minutes exposure (SGF, pH 2.5) all the isolates were at a survival level of ≤2%. In contrast, the virulent isolates survived at this level of acidity when suspended in PBS (pH 2.5). Increased survival was observed in SGF (pH 4.0) with all the virulent isolates being characterized as acid resistant. For all strains, exposure to SGF (pH 2.5) was significantly different (*P *< 0.001) from SGF (pH 4.0) and PBS (pH 2.5). Differences were observed between SGF (pH 4.0) and PBS (pH 2.5), but they were not statistically significant (*P *= 0.062). The two attenuated strains (KC 1482 and LVS) were less resistant under all conditions.

### Acid tolerance after storage in water

The effect of overnight storage in sterile creek water on acid resistance is shown in Table 2. The strains in the creek water decreased by <1.0 log_10_ CFU/mL during storage (data not shown). Cells of the virulent strains stored at low-nutrient conditions demonstrated a mean 150-fold greater resistance compared to the nonnutrient stressed cells when exposed to SGF (pH 2.5) for 120 min. Although these differences were statistically significant (*P *< 0.001) for each strain, the response of the stressed cells was below the 10% survival level criterion. Prior exposure in creek water did not change the response for the LVS.

**Table 2:** Survival of *Francisella tularensis* ssp. *holarctica* nutrient stressed in sterile creek water prior to exposure in synthetic gastric fluid, pH 2.5.

Isolate	Mean % survival after 120 min in synthetic gastric fluid^1^	
	Nonstressed cells	Stressed cells
IN99	0.023	3.15^2^
NY98	0.004	1.02^2^
OR96	0.007	0.40^2^
LVS	<0.001	<0.001

LVS, live vaccine strain.

1 Initial inoculum in synthetic gastric fluid ranged from 4.0 to 6.2 log_10_ CFU/mL.

2 Stressed cells survived at significantly higher rate than nonstressed cells, *P* < 0.001.

## Discussion

The observed low survival in SGF (pH 2.5) was in sharp contrast to the extreme acid resistance seen in PBS (pH 2.5). Experimental conditions where stationary-phase cultures are exposed to PBS (pH 2.0) at 37°C have previously been used for determining acid resistance (DeKoning-Ward and Robins-Browne 1995; Tennant et al. 2008). The present results are consistent with in vitro acid tolerance data in saline reported for a virulent strain of *F. tularensis* (KuoLee et al. 2007). Most studies have considered hydrochloric acid to be the primary bactericidal agent in the stomach. The current results support the findings of Zhu et al. (2006) indicating a synergistic effect of low pH and digestive enzymes which was not observed under low-pH conditions alone. The entrance of food into the stomach leads to a transient elevation in gastric pH. When the pH of the SGF was increased to a level common after the ingestion of a meal, the organism demonstrated a higher level of survival, suggesting that consumption of contaminated food may increase the likelihood of gastrointestinal infection. These findings indicate that exposure to acidified SGF (pH 2.5) may represent a more realistic condition for determining the capability of the organism to survive gastric passage under fasting conditions.

The level of acid resistance of a pathogen has been suggested to correlate with the oral infectious dose required to cause disease. A low infectious dose has been linked to the ability to tolerate acidic pH levels typical of the range found in gastric fluid (Gorden and Small 1993). The observed inability of *F. tularensis* to survive in SGF at pH 2.5 is in keeping with studies showing that the oral infectious dose is quite high. Hornick et al. (1967) reported an oral ID_50_ of 10^8^ when human subjects were challenged using capsules containing the SchuS4 strain. When suspensions of the organism were gargled, a much lower ID was required to produce the oropharyngeal form of the disease. Similar ID_50_ levels (circa 10^7^) have been reported by the same group for oral challenge studies with primates (Tulis et al. 1969). Lethality studies using mice reported a *F. tularensis* 50% lethal dose (LD_50_) by oral challenge of approximately 10^6^ (Quan et al. 1956; KuoLee et al. 2007). Although these are relatively high threshold doses and gastrointestinal tularemia is rare, this form of the disease does occur and may be associated with ingestion of highly contaminated food or water. It should be noted that gastrointestinal symptoms can also result from tularemia acquired through other transmission routes, such as tick bites (Zaidi and Singer 2002). Several studies have also indicated that protozoa might serve as an important environmental reservoir of *F. tularensis*, prolonging its persistence in water (Berdal et al. 1996; Abd et al. 2003; El-Etr et al. 2009).

The use of attenuated strains as surrogates for virulent *F. tularensis* facilitates research efforts by eliminating the exacting biosecurity and biosafety conditions (BSL3) required for experimentation with virulent strains. In this study the attenuated strains did not prove to be good surrogate organisms. The reason for this finding remains unclear, but may be due to differences in cell wall and capsular composition. Ultrastructure studies have revealed that vaccinal and avirulent strains have a thinner capsule than virulent strains (Gerasimov et al. 1997). The lesser amount of capsular material has been hypothesized to be responsible for the increased susceptibility of an attenuated strain to killing during phagocytosis (Löfgren et al. 1983). In a genome comparison study, Rohmer et al. (2006) noted a mutation in a glycosylation transferase gene in the LVS which potentially could alter cell wall lipopolysaccharide and capsular composition. Similar findings regarding decreased resistance to chlorination (O'Connell et al. 2011) and exposure to detergents (Chalabaev et al. 2011) have been reported. The LVS has also been shown to be sensitive to low-pH conditions when incubated in an acidified growth medium (Meibom et al. 2008; Dieppedale et al. 2011). The avirulent KC 1482 exhibited a similar response as the LVS. This strain has been reported to be one of the most divergent strains examined in a genome-wide, microarray-based analysis of the genus (Broekhuijsen et al. 2003). Such divergence could likely alter phenotypic characteristics which could influence the response to acidic exposure.

In an attempt to partially mimic the conditions of a natural aquatic environment which the organism might encounter prior to ingestion, isolates were stored overnight in water (Bodmer et al. 2000). Incubation under low-nutrient conditions is more consistent with the natural oral exposure route in water than the more common preadaptation procedures used for enteric bacteria where organisms are exposed to a moderately low pH similar to ingestion of slightly acidic food. Resistance produced by low-nutrient conditions has been shown to be as protective as other forms of preadaptation (Siegele and Kolter 1992). The observed significant difference seen between the nutrient stressed and nonstressed cells is noteworthy. Based upon these findings further research would be warranted to address the effects of low-nutrient preadaptation for other conditions such as exposure to oxidants used in water treatment.

The current results indicate that *F. tularensis* under certain conditions can survive at a very low pH, but the organism is sensitive to in vitro conditions similar to those found in the fasting human stomach. This may help explain the apparent contradiction of an acid-resistant organism requiring a very high ID to cause infection via oral exposure. These results further support clinical reports where the oropharyngeal form of tularemia has been shown to be the primary disease manifestation when the organism is acquired by ingestion of contaminated water.
